# Highly Engaged Video-Watching Pattern in Asynchronous Online Pharmacology Course in Pre-clinical 4th-Year Medical Students Was Associated With a Good Self-Expectation, Understanding, and Performance

**DOI:** 10.3389/fmed.2021.799412

**Published:** 2022-01-21

**Authors:** Jann-Yuan Wang, Chia-Hsien Yang, Wei-Chih Liao, Kai-Chien Yang, I-Wen Chang, Bor-Ching Sheu, Yen-Hsuan Ni

**Affiliations:** ^1^Division of Curriculum Integration, Center of Faculty Development, National Taiwan University College of Medicine, Taipei, Taiwan; ^2^Department of Internal Medicine, National Taiwan University College of Medicine, Taipei, Taiwan; ^3^Office of International Affairs and Global Master of Business Administration Program, National Taiwan University College of Management, Taipei, Taiwan; ^4^Department and Graduate Institute of Pharmacology, National Taiwan University College of Medicine, Taipei, Taiwan; ^5^School of Medicine, National Taiwan University College of Medicine, Taipei, Taiwan; ^6^Department of Obstetrics and Gynecology, National Taiwan University College of Medicine, Taipei, Taiwan; ^7^Department of Pediatrics, National Taiwan University College of Medicine, Taipei, Taiwan

**Keywords:** engagement, learning analytics, learning outcome, medical education, online video-based learning, video-watching pattern

## Abstract

**Background:**

Online video-based learning is more common in higher education. Investigating students' viewing behaviors while watching online video lectures is essential for instructors to understand their learning status so that the course content, structure, and media selection can be improved continuously. The current study identified the engagement level of the learners based on their online video-watching behaviors, and tested the correlation between the engagement level and learning outcome.

**Methods:**

The action logs of watching online video lectures in 2020 Spring Pharmacology of the 4th-year medical students of the 6-year course and their feedbacks by questionnaires after each exam during the semester were provided anonymously. The data were analyzed and visualized for an efficient way to comprehend and interpret. To define the student's engagement level in his or her video-based learning journey, three viewing criteria, “*Completion*,” “*Pausing*,” and “*Repeated watching*” were identified. We evaluated the association between the engagement level and the students' learning outcomes, including their learning satisfaction, knowledge acquisition progresses based on assessment results, and the grades measured by the instructors.

**Results:**

The graphs and the charts demonstrate whether the students allocated enough time to finish the video lectures (completion), paused for a while, then resumed the video (pausing), or replayed the specific sections of video content (repeated watching). The engagement level with video lectures, evaluated by pre-defined thresholds for “*Completion*,” “*Pausing*,” and “*Repeated watching*” had a positive correlation with the learning outcomes.

**Conclusions:**

We suggested that an engagement dashboard containing real-time visualized information on students' online video-watching behaviors can be developed to help instructors to monitor students' learning progress and improve teaching in a timely fashion. It can also help each student to re-feel the stimulation of peers, prompt self-monitoring, improve their learning attitudes and disciplines for better learning outcomes. This innovative way of assessing student's engagement during online video-based learning can also be used for quality assurance purposes.

## Introduction

Higher education has increasingly developed and offered online courses as part of their academic curriculum in recent two decades (1–3). It reaches a wide range of audiences and improves teaching and learning environments (4, 5). In an asynchronous online course, the time-independent delivery mode respects student's autonomy and has benefited students with different paces and learning strategies (6, 7). Although students can learn at their own pace freely, they are required to be self-disciplined for effective learning (8).

As online courses are more and more common, students often report difficulties in attention and commitments to their online courses (9). There is an increasing concern with regards to students' lack of persistence and engagement, reflected by low activity (10, 11) and high dropout rate compared (12–15) with traditional in-person courses. Previous studies demonstrated that lack of persistence, reflecting low engagement and poor self-management, is an essential factor leading to attrition among students in online courses and suboptimal academic achievements (11, 16, 17). Therefore, instructors need to track online learners' engagement status to ensure teaching effectiveness.

Unlike in-person or hybrid courses, online learning does not involve physical interaction between students and instructors (18). Research in learning analytics has revealed that providing a real-time dashboard to check online learning progress and participation level compared with peers can support students' self-management and facilitate their learning attitudes to improve their comprehensions and learning achievements (19). Therefore, tracing and analyzing the online learning behaviors presents an effective solution for students to manage their learnings better, which reduces the instructor's workloads in class management.

Assessment of the online video learning process is even more crucial for instructors, given that it may serve as a means of understanding students' involvement and engagement with the course materials (20). Such information generated by well-designed learning analytics may benefit instructors in several ways, including (1) understanding students' involvements and performances, (2) improving instruction and assessment in real-time, (3) modifying online teaching materials to accommodate students' needs, (4) adjusting teaching styles to raise students' interests and facilitate persistence, and (5) tracking students' usage of different parts of a learning website to have a better understanding with regards to their learning processes, effectiveness, and suitability (21–23). In other words, the ability to understand students' online video-based learning activities and evaluate teaching performance is indispensable and helpful for instructors to design online courses and support their students (24).

To brace for digital education, we started to discuss and re-design some courses for online video-based learning starting from 2019. However, in 2020 Spring, the required Pharmacology course, initially taught in the classroom with more than 150 pre-clinical medical students (4th year of the 6-year course), was changed to be online video lectures under the COVID-19 crisis. Though the COVID-19 outbreak pushed us moving faster, it also revealed opportunities for teaching innovations.

However, as we adapted online video-based learning to the COVID-19 situation, we experienced challenges in understanding the students' real-time learning statuses without in-person interactions like we usually do in the classrooms. Although we still had measurements to grade the students' learning results, we were considering a better solution to know their learning progresses so that we would have references to enhance the course content, media selection, and the lecturing style in the future.

Therefore, we analyzed the video viewing logs, delimited completion, pausing, and repeated watching as the engagement criteria, and tested the feasibility of defining three levels of engagement with video lectures. The correlation between engagement level and learning outcome regarding the learner's satisfaction and performance was also investigated.

### Hypothesis

We hypothesized that the engagement level of online video watching can be measured by combinations of learning analytics and may correlate with learning outcome.

### Study Aim

This study aimed to investigate whether the undergraduate senior medical students' online video-watching engagement levels, measured by their viewing action logs, were associated with their online Pharmacology learning outcome. A real-time engagement dashboard can be further developed to facilitate online video-based learning and teaching.

### Research Questions

1. How can we define completion, pausing, and repeated watching by analyzing the online Pharmacology video viewing logs?
2. How can we use completion, pausing, and repeated watching to determine the engagement level?
3. Does the highly engaged learning lead to a better learning outcome in the online pharmacology video lectures?

## Research Context and Learning Management System

Though the movement of Pharmacology course from in-person to online video lectures seemed to be a forced shift resulted from the COVID-19 pandemic, the video lectures have been used as part of the teaching materials to improve learning effectiveness before the COVID-19 crisis, based on the advice from the Curriculum Committee at our institution, the National Taiwan University College of Medicine (NTUCM) and the suggestions from students, and alumni.

### Research Context

The research context for this study was a Pharmacology course offered for the pre-clinical senior medical students (4th year of the six-year course) at NTUCM, a top medical school in Taiwan. The course was mandatory for medical students and not open to other majors as an elective course. The course content was delivered by 20 video lectures online asynchronously (9 and 11 video lectures in the first and second half of the course, respectively), delivered by 10 teachers. Each video was around 60 min and uploaded weekly to the National Taiwan University COurses OnLine (NTU COOL) system with the related course materials. The Curriculum Committee and the course instructors define the learning route that drives the students to reach proficiency in the semester. The students could arrange their online learning on their own.

Since the students learned from the videos during the outbreak, the instructors could use the reserved in-person class times to foster learning. Before the original in-class times, the teaching assistant would collect the students' questions, feedback, and comments posted on the NTU COOL system. According to the students' learning needs, the teaching assistant discussed with the related instructors the online workshop itinerary (independent beyond the online video-learning courses) for question clarifications or further discussions. If nothing were posted, the teaching assistant would consult with the associated instructors whether the students are in fear of appearing foolish, having gaps in knowledge, or hard to form the question. Based on the previous teaching experiences or feedback from the alumni, the associated instructors would guide the teaching assistant to conduct an oral quiz to reflect students' learning or offer Supplementary Materials to enhance students' understanding in the workshop. Otherwise, the associated instructors might suspend online workshops one time, regardless of whether they considered students had engaged and learned. The learning outcome would then be evaluated by mid-term and final exams.

The students were required to take four online exams and fill out the learning satisfaction questionnaire after each exam. The teaching assistants maintained the syllabus, announced updates, or posted reminders on the course bulletin board over the Collaborative Enhanced Instruction By Asynchronous (CEIBA) course system to ensure all the students were on the same page.

### Learning Management System

Currently, NTU provides two platforms to manage course delivery, CEIBA and NTU COOL. CEIBA links up with educational administration systems to assist faculty members in creating courses websites, by which instructors can upload courses handouts, announce assignments, and release academic grades. It also provides other functions such as forums, etc., to support the teaching and learning of the courses. Although CEIBA fulfills the fundamental teaching requirements, it is a legacy system without supporting online video-learning.

In recent years, many high educational institutions started developing web-based learning in either a hybrid or a purely online mode before the COVID-19 outbreak. The online learning strategy enables educational institutions to implement a learner-centered approach to teaching where learners are given space and flexibility to indulge in constructive learning activities (5, 25, 26). It can lessen teacher's workload, increase the flexibility and diversity of course designs, support students to manage their own pace of learning, and foster students' autonomy.

To brace for online learning, NTU initiated a learning management platform, the NTU COOL, in 2017, which went live officially in 2019, with the following main features:

1. Fundamental LMS (Learning Management System) Functions: NTU COOL has all necessary functions to support learning in NTU, including the curriculum organization and delivery, course material management, discussion board, learner assessment, and peer evaluation.
2. Video-based Learning: It allows users to upload their videos or to import videos from YouTube. Students can play videos at different speeds and jump to specific segments accordingly to their learning needs. When the network bandwidth is limited, the video resolution can be adjusted accordingly. Both instructors and learners can post comments and reply to each other on specific parts of videos.
3. Interaction Environment: In addition to the bulletin board or discussion board, instructors can set up different forums by subject or group to facilitate teaching effectiveness. Recently, a new feature, named “Symphony,” was launched for users to exchange ideas. Teachers can release text files (PDF) onto the system, and students can mark on the languages to comment or clarify their questions.
4. Tracking and Report: NTU COOL visualizes some learning data of students, including their video-viewing time, activity participation records, and learning progresses. Those graphs are valuable tools for both instructors and students to understand their learning situations.

Since NTU COOL is more advanced and certified by ISO 27001, the Office of Academic Affairs has decided to stop the service of CEIBA on August 1, 2022, after several years of operation in parallel to ease user anxiety and reduce replacement risk.

Detailed introduction and usage of the NTU COOL system are available on the website (https://www.dlc.ntu.edu.tw/en/coolsupport/). A series of screenshots have been added in the online Supplementary File to illustrate the NTU COOL learning platform (Supplementary Figure 4 in the Supplementary File). The videos were Microsoft PowerPoint slideshow with the instructor's audio narration. These were uploaded to the integrated organ system-based online scaffolding learning modules in the NTU COOL system for students' convenience to access and view.

When the COVID-19 outbreak happened, in-person classes of more than 60 students at NTU had to be distance learning, according to the guidance announced by the Central Epidemic Command Center (CECC) of Taiwan Centers for Disease Control (TCDC). As of more than 150 students, the face-to-face Pharmacology class was soon replaced by the online video lectures and virtual workshops for question clarifications or group discussions since April 6th, 2020 (2nd semester).

## Learning Analytics of Video-Watching Behavior in Asynchronous Online Video Learning

Asynchronous online video learning is a self-regulated learning process involving systematic efforts, including planning, conducting, regulating, and evaluating to attain learning goals (27, 28) to transform the learner's mental abilities into academic skills (29). Since 1960, educational videos have been used as learning aids in medical education (30). Though online video learning has become a feasible and popular learning module that can be applied to clinical medicine (31), its success in medical education requires learners' self-regulated learning and active engagement (32). In general, online video-based learnings are more autonomous than traditional face-to-face or blended courses (33). Therefore, it is vital to identify prominent characteristics or strategies leading to successful learning in asynchronous online video-earning environments (34). Moreover, understanding the video-watching behaviors of students over time is crucial to provide timely instructional support for improving both teaching and learning. Previous studies have identified and validated several attributes of asynchronous online video learning, detailed in the following.

### Time Investment

Asynchronous online courses require students to study on their own without the instructor's direct supervision. Therefore, time spent on video content of asynchronous online courses is a fundamental issue in understanding course topics and successful learning (35). Having sufficient time is particularly important in asynchronous online courses because learning time depends entirely on students' perceptions of the importance of the study and students' decisions and abilities to secure and allocate time for learning activities (36). Time spent is thus regarded as one of the fundamental dimensions of engagement and has been validated to positively impact students' achievements, final grades, and retentions in the online courses (37, 38). Specifically, imperative attributes for time spent may include the total number of online sessions completed (4, 22), hours viewing main content (22, 39), time spent in each week (35, 40), and time spent on learning tools (27).

### Pausing and Repeated Watching

During online video learning, in order to understand better what has been taught in the video, learners may either pause the lecture to take a break, to think and reflect, to write a synthesizing note on the content, or to collaborate with a peer, or to hover over the filmstrip to locate a specific piece of information to navigate and repeat explanations until they are fully understood (40). In a study investigating the underlying meaning of activities during online video learning with ViDeX, Seo and his colleagues found that during exam week, students “*Search*” more and “*Reflect*” less, probably in an attempt to be more efficient and mindful in their time in seeking video segments that they perceive are valuable for the exam (40). The way of time utilization is another sign of strategic engagement in online video learning, implying that students selectively pick parts that they consider vitally important to re-watch (41, 42).

### Study Regularity and Timing

Study regularity has also been acknowledged as a strong indicator of self-regulated learning in asynchronous online courses, representing learners' earnestness and engagement in this learning environment. These self-regulated learners work hard, regularly allocate their time and, overcome obstacles on the way forward (43, 44). In a study analyzing log data from 284 undergraduate students in an asynchronous online statistics course, Kim et al. noted that self-regulated learners were more likely to study regularly and demonstrated consistent commitment to their learnings, leading to significantly higher final grades (35).

In the previously mentioned study done by Seo and his colleagues (40), the authors found that the video viewing behavior in exam weeks (the one week before students take the exam) was different from that in non-exam weeks. Students “*Clarify*” less during exam weeks probably because the activity of “*Clarify*,” such as rewinding and reducing playback speed, increases engagement time with the video. Usually, this activity implies that learners intend to understand information comprehensively or to clarify video segments. Specifically, content learning no later than two weeks before exams significantly increases the probability of being a self-regulated learner and implies that the learner engages in content learning with sufficient time. In contrast, students who studied right before or during the exam week are likely to have a learning pattern of cramming (29).

Learning analytics is a significant asset for discovering crucial information and knowledge from an education setting to assess learning effectiveness and improve teaching (45, 46). In this study, the learners' anonymous video-viewing data and course feedback were collected and analyzed. The parameters were developed to generate various attributes of viewing behaviors, which were visualized and used to define engagement levels with online Pharmacology video lectures.

## Methods

First, we analyzed the students' video viewing logs to identify the parameters of time investment, the situation of pausing and repeated watching, learning regularity, and watching timing. The associated parameters determined three key viewing behaviors (completion, pausing, and repeated watching) and evaluated their video usage's time investment and regularity. Secondly, we defined their online video learning engagement level (high, intermediate, or low) based on the composition of the three key viewing behaviors. Lastly, the student's learning satisfaction, self-assessment of learning effectiveness, and grades were compared among students with different engagement levels.

### Participants

Initially, a total of 155 pre-clinical medical students (4th year of 6-year course) were enrolled in the course. Three of the 155 students were repeating the course for a second time, and one student neither finished the questionnaires after the exams nor provided his feedback during the whole semester. Therefore, we used the data from a total of 149 students for further analyses. The 149 participants were all 4th-year medical students of the 6-year course. Among them, 111 (74.5%) were male. The students were informed that completion of questionnaires was compulsory but would not be accounted for grading.

### Data Collection and Processing

Students' video-viewing data (action logs), grades, course feedback, and questionnaires data as well as their personal information were stored in the Digital Learning Center, National Taiwan University (NTM-DLC). After the Institutional Review Board (IRB) approved our research proposal, the de-identified data (anonymous to the users) in CSV (comma-separated values) file was provided by the Digital Learning Center, National Taiwan University (NTU-DLC) after the course was completed. For the convenience of analysis, the first two and the last two exam scores were averaged separately to be used as their exam results for the first and the second half-semester, respectively.

**Figure 1:**
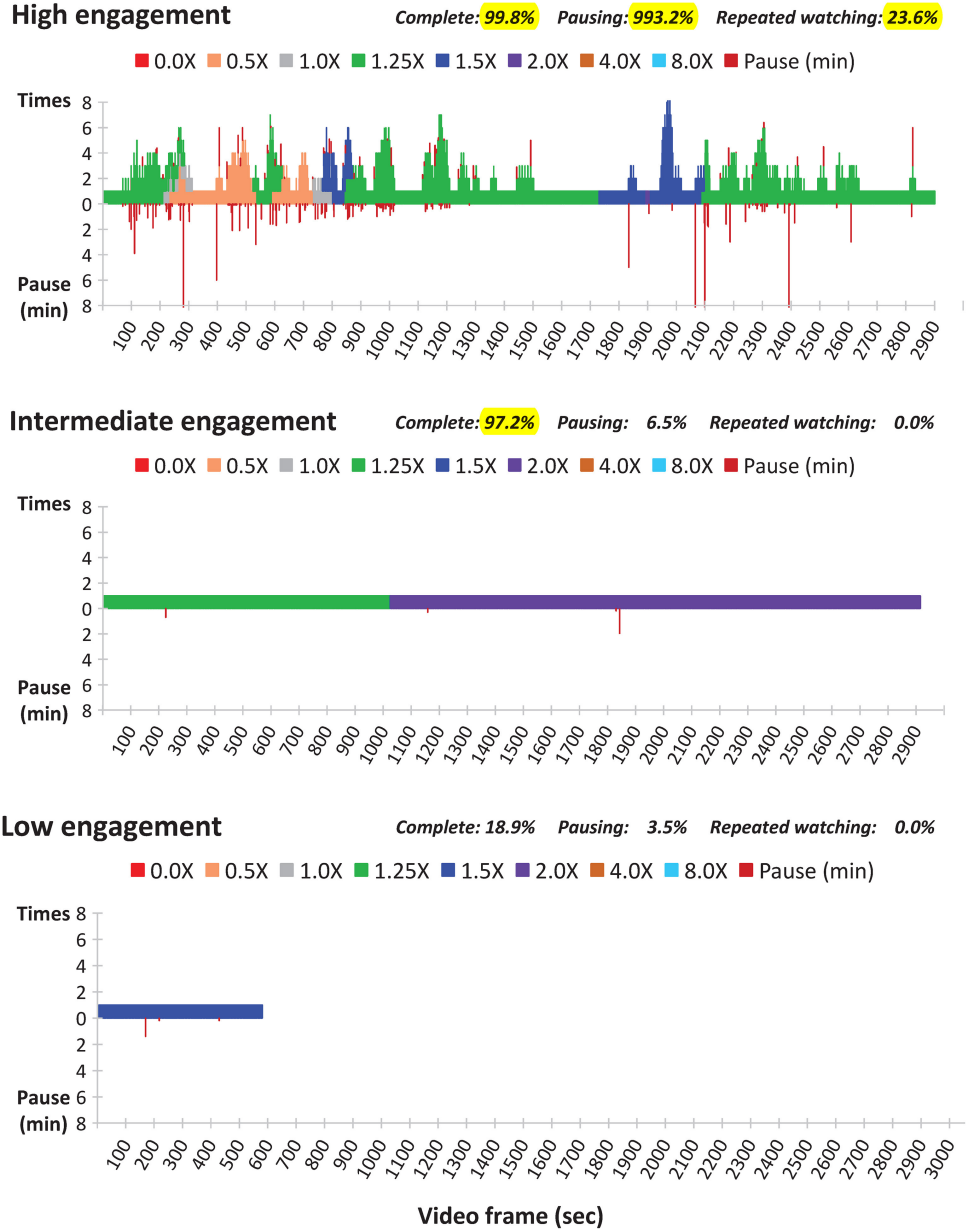
Examples of high engagement (upper panel), intermediate engagement (middle panel) and low engagement (lower panel) with video-based learning (X: play rate).

JYW processed the raw data of action logs of each participant using Microsoft Access. The action logs were visualized to express watching speed, rewinding, repetition, and pausing. Furthermore, three online video-viewing behaviors, completeness, pausing, and repeated watching (Figure 1) were identified (23).

In this study, a threshold for a parameter of video-watching behavior was initially selected by the best dichotomization for correlation with grades. In cases that two cutoff points performed equally well, the threshold was further determined by the consensus of JYW, CHY, and IWC.

The questionnaires were initially created by a committee, which consisted of the program directors from course-related departments, two pre-clinical senior medical students, and four senior tutors and one teaching assistant from the Center of Faculty Development (CFD) of NTUCM in 2010 for assessing the quality and efficacy of teaching, rather than for research purpose. The questionnaires have been used for years and revised several times. Five key questions in the questionnaires assessing students' learning expectation, satisfaction, and efficacy by using 5-point Likert scale system (strongly disagree, disagree, neutral, agree, strongly agree) (47) were analyzed in the study (Supplementary Table 1 in online Supplementary File).

### Definition of Parameters

A total of 11 parameters (Table 1) were used to measure the students' online video-watching behaviors in this study, including five for time investment, one for pausing, one for repeated watching, and four for regularity and timing.

1. Time investment:
  a) The finished rate, “the watched video length (excluding re-watch) divided by the video length” (unit: %), was the percent of the video the audience watched.
  b) The watch rate, “the watch time (including re-watch) divided by the video length” (unit: %), was performed to understand how much more or less time the students were watched compared to the total video length.
  c) The engaged-view rate, the percentage of videos viewed in high engagement level (unit: %), was to understand the high engagement situation of the class as a whole. The definitions of engagement levels are stated in Section 4.6.
  d) The initial watch rate, “sum of each initial video-watching time divided by the total length of all videos” (unit: %), was to know the students' learning activation status. The “initial” referred to the first time seeing a video.
  e) The initial learning progress, “the sum of each initial video-watching time divided by the total video-watching time” (unit: %), was developed to understand the students' initial learning progress compared to their total watching time. The “initial” referred to the first time seeing a video.
2. Pausing:
  The pause rate, “total paused duration divided by total video length” (unit: %), was computed to understand how long a student paused the videos during online video-watching.
3. Repeated watching:
  The re-watch rate, “the sum of video seconds watched for three or more times divided by total video length” (unit: %), gave us an overview regarding the proportion of the video length being repeatedly watched.
4. Regularity and timing: The following measurements were determined for each student to investigate their learning regularity and timing.
  a) Learning days between two exams: number of days in watching videos between two exams (unit: day).
  b) When to start learning right after an exam: how many days after an exam to start watching the video (unit: day).
  c) The view rate before exam week: the number of exam videos viewed before exam week divided by the total number of exam videos (unit: %) was performed to learn their exam preparation activation status before exam week.
  d) The initial finished rate before exam week: “the sum of the initial video watch length (excluding re-watch) between two exams and before exam week divided by the total length of the watched videos before exam week” (unit: %), was used to understand their exam preparation progress.

**Table 1:** Learning analytics metrics glossary and definition.

**Terms**	**Equation/Definition**	**Measurement in the scenario**
Finished rate (%)	$\frac{\text{Total video length been watched}}{\text{Total video length}}$	270/540 = 50.0%
Watch rate (%)	$\frac{\text{Total video}-\text{watching time}}{\text{Total video length}}$	360/540 = 66.7%
Initial watch rate (%)	$\frac{\text{Sum of each initial video}-\text{watching time}}{\text{Total video length}}$	180/540 = 33.3%
Pause rate (%)	$\frac{\text{Total paused duration}}{\text{Total video length}}$	10.8/540 = 2.0%
Re-watch rate (%)	$\frac{\text{Video length been watched}\geq 3\text{times}}{\text{Total video length}}$	5.4/540 = 1.0%
Engaged-view rate (%)	$\frac{\text{No}.\text{ of videos been watched in highly engaged mode}}{\text{Total No}.\text{ of videos}}$	1/9 = 11.1%
Initial learning progress (%)	$\frac{\text{Sum of each initial video}-\text{watching time}}{\text{Total video}-\text{watching time}}$	180/360 = 50.0%
Learning days between two exams (day)	*No*. *of days in watching videos between two exams*	8/50 = 16.0%
When to start learning right after an exam (day)	*How many days after an exam to start watching the video*	15
View rate before exam week (%)	$\frac{\text{No}.\text{ of videos been initially watched before exam week}}{\text{Total No}.\text{ of videos between }2\text{ exams}}$	3/9 = 33.3%
Initial finished rate before exam week (%)	$\frac{\text{Video length been initially watched before exam week}}{\text{Total length of videos been initially watched before exam week}}$	45/180 = 25.0%

*The table below explains the terms we used in this study. Following is the scenario to provide a case example. There are nine 60-min videos in the first half of the course. A student started watching the video on the 15th day of the semester. In eight days, he spent 360 min watching 5 videos. Among these 5 watched videos, only one was viewed at a high engagement level. In addition, the watched length of these 5 videos is 270 min and the sum of the first watch time in these 5 videos is 180 min. In the 360-min watch time, a total of 10.8 min were paused. During the 270-min watched video length, there were 5.4-min re-watched more than three times. Before exam week, 3 videos were viewed. The sum of the first watch time for those 3 videos is 45 min. The table below is his video-watching measurements. (Abbreviation: No., number)*.

### Definition of Engagement Level

Video learning engagement is related to students' time investment and interactions with the video content. Therefore, we used the finished rate, the pausing rate, and the re-watch rate to identify three key watching behaviors, “Completion,” “Pausing,” and “Repeated Watching.”

“***Completion***” was defined as the finished rate >90%, “***Pausing***” was defined as the pausing rate ≥10%, and “***Repeated watching***” was defined as repeated watching rate >3%. A video-watching status was considered highly engaged in the presence of 2 or 3 attributes, intermediately engaged in the presence of only one attribute, and low engaged in the absence of all 3 attributes.

### Statistical Analysis

Quantitative data were expressed in percentages where appropriate. Non-parametric tests were employed to compare group differences. Categorical variables were compared using the *chi*-squared test, whereas Mann–Whitney *U* and the Kruskal–Wallis tests were employed to compare the difference in continuous variables for 2 and 3 groups, respectively. The Pearson correlation coefficient assessed the correlation between two variables. A two-sided *p* < 0.05 was considered statistically significant. Statistical analyses were performed using SPSS version 21.0 (SPSS Inc., Chicago, IL, USA).

## Results

Among the 149 students, 46 (30.9%), 42 (28.2%), and 61 (40.9%) students were considered as highly engaged, intermediately engaged, and low engaged in the first half of the course; while during the second half of the course, 30 (20.1%), 48 (32.2%), and 71 (47.7%), respectively. The engagement status in the first and the second half of the online Pharmacology course was significantly correlated (Pearson correlation coefficient = 0.557) (Table 2).

**Table 2:** Correlations between the engagement status in first and second half of Pharmacology course (Pearson correlation coefficient = 0.557).

		**Engagement status in second half of Pharmacology course**		
		**Low**	**Intermediate**	**High**
Engagement status in first half of Pharmacology course	Low	47 (31.3%)	11 (7.3%)	4 (2.7%)
	Intermediate	18 (12.0%)	21 (14.0%)	3 (2.0%)
	High	7 (4.7%)	16 (10.7%)	23 (15.3%)

The illustrations of high, intermediate, and low engagement with online video-watching were shown in Figure 1. Unlike the latter two, the high engagement composed 2 or 3 viewing behaviors of completion, pausing, and re-watching.

### Online Video-Watching Behaviors in Different Engagement Status

**Figure 2:**
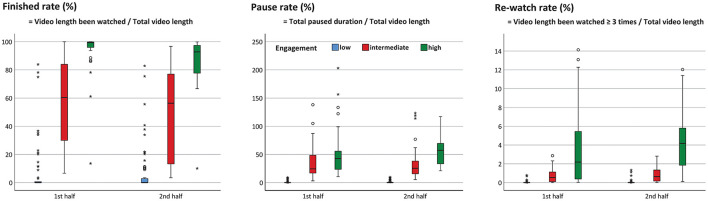
The differences in the finished rate, the pause rate, and the re-watch rate among the students with different engagement levels (*p* < 0.001 for all). *represents extreme value; °represents potential outlier.

The finished rate was significantly different among participants with high, intermediate, and low levels of engagement in the first half (median: 99.2 vs. 60.5 vs. 0%, *p* < 0.001) and the second half (median: 92.8 vs. 56.4 vs. 0%, *p* < 0.001) of the online course (Figure 2, left panel). Similarly, the pause rate was significantly different among the 3 groups of participants in the first half (median: 42.8 vs. 24.4 vs. 0%, *p* < 0.001) and the second half (median: 57.4 vs. 25.4 vs. 0%, *p* < 0.001) of the course (Figure 2, middle panel). The re-watch rate was also different among the 3 groups in the first half (median: 2.2 vs. 0.5 vs. 0%, *p* < 0.001) and the second half (median: 4.2 vs. 0.6 vs. 0%, *p* < 0.001) of the course (Figure 2, right panel).

**Figure 3:**
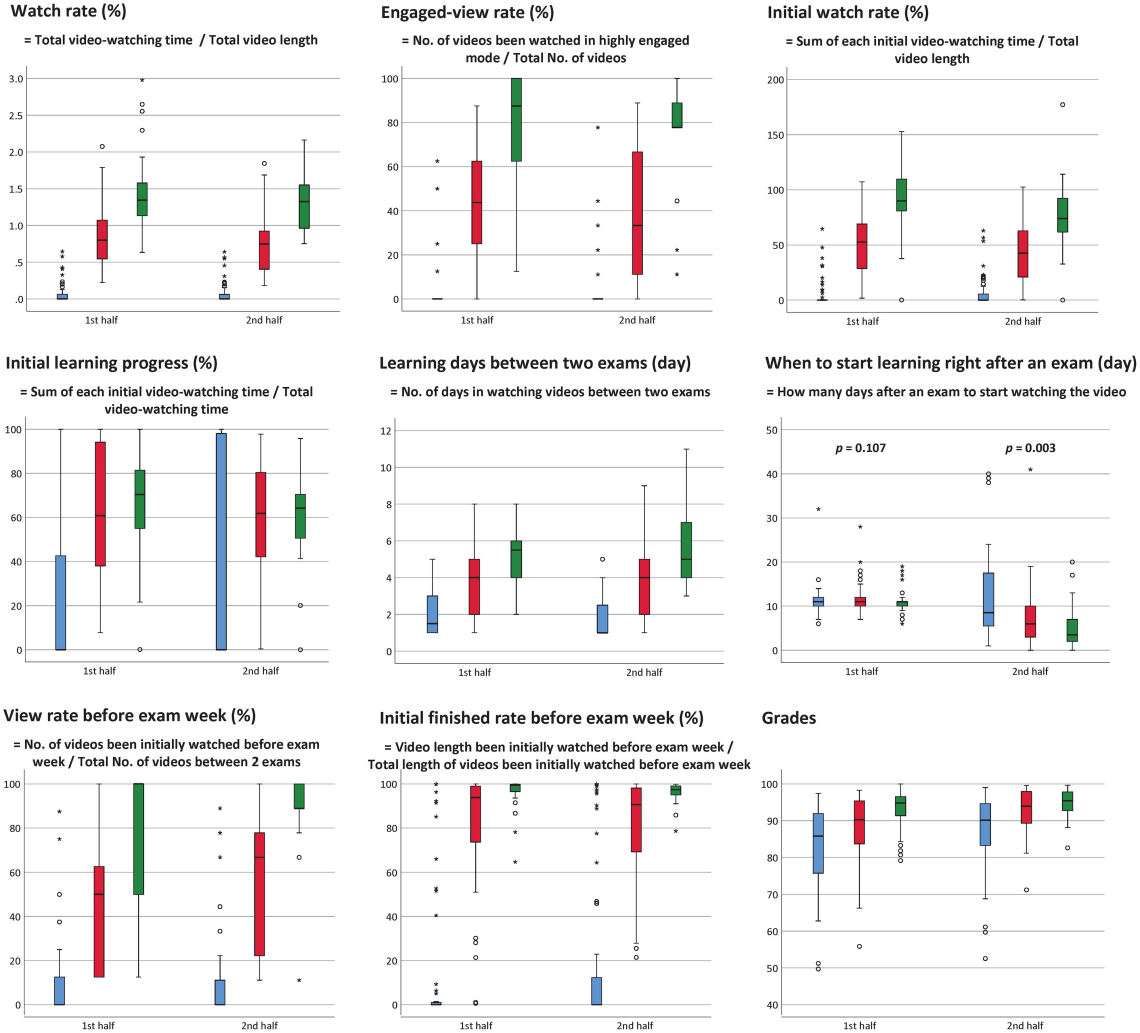
Online video-watching characteristics among the 3 groups with different engagement levels (*p* < 0.001 for all unless otherwise mentioned) (Abbreviation: No., number). *represents extreme value; °represents potential outlier.

Other characteristics of online video-watching among the 3 groups with different levels of engagement were shown in Figure 3. Statistical analyses revealed that the watching rate (Figure 3, left upper panel), the engaged-view rate (Figure 3, upper center panel), the initial watch rate (Figure 3, right upper panel), and the initial learning progress (Figure 3, left middle panel) (all *p* < 0.001) of highly engaged participants were higher than the other two groups.

The highly engaged group spent more learning days between two exams (Figure 3, center panel) during the whole semester, began to watch the videos earlier than the other two groups in the second half (*p* = 0.003) of the course, but had no difference in the first half (*p* = 0.107) of the course (Figure 3, right middle panel). The highly engaged group also had a higher view rate before exam week (Figure 3, left lower panel) and a better initial finished rate before exam week (Figure 3, lower center panel) (all *p* < 0.001).

### Engagement Status and Learning Outcome

Statistical analysis showed that the exam grades were significantly different (*p* < 0.001 in both the first and second half of the course) among the 3 groups with different levels of engagement. Not surprisingly, the high-engaged group had the best exam result (Figure 3, right lower panel).

Results of survey questionnaires were shown in Supplementary Table 2 in online Supplementary File. For each of the five questions, <5% of students reported either disagree or strongly disagree.

In the first half of the course, the high engagement was associated with better learning outcomes, including a higher self-learning satisfaction and a better understanding of the underlying concepts. In addition, the students who learned in high-engaged mode expressed that they could connect Pharmacology with other classes more efficiently and used what they had learned from Pharmacology to construct the understandings of other subjects more widely (Table 3). Though the findings were similar in the second half of the course, the differences failed to reach statistical significance except for understanding underlying concepts.

**Table 3:** Analytic results of questionnaires on learning efficacy and satisfaction.

	**First half of the online Pharmacology course**							**Second half of the online Pharmacology course**						
	**Low engagement (*****n*** **= 61)**		**Intermediate engagement (*****n*** **= 42)**		**High engagement (*****n*** **= 46)**		***P***	**Low engagement (*****n*** **= 71)**		**Intermediate engagement (*****n*** **= 48)**		**High engagement (*****n*** **= 30)**		***P***
	**No**	**Yes**	**No**	**Yes**	**No**	**Yes**		**No**	**Yes**	**No**	**Yes**	**No**	**Yes**	
Does Pharmacology meet your learning expectations?	47 (77%)	14 (23%)	25 (60%)	17 (40%)	20 (43%)	26 (57%)	**0.002**	42 (59%)	29 (41%)	25 (52%)	23 (48%)	12 (40%)	18 (60%)	0.209
Do the organized course videos help you understand the underlying concepts?	32 (52%)	29 (48%)	13 (31%)	29 (69%)	5 (11%)	41 (89%)	**0.000**	34 (48%)	37 (52%)	9 (19%)	39 (81%)	7 (23%)	23 (77%)	**0.002**
Are you fine with the difficulty level?	18 (30%)	43 (70%)	9 (21%)	33 (79%)	6 (13%)	40 (87%)	0.126	16 (23%)	55 (77%)	11 (23%)	37 (77%)	2 (7%)	28 (93%)	0.140
Can you connect Pharmacology with other classes?	17 (28%)	44 (72%)	7 (17%)	35 (83%)	4 (9%)	42 (91%)	**0.039**	15 (21%)	56 (79%)	11 (23%)	37 (77%)	2 (7%)	28 (93%)	0.159
Can you use what you've already learned from Pharmacology to construct the understandings of other subjects?	18 (30%)	43 (70%)	9 (21%)	33 (79%)	5 (11%)	41 (89%)	**0.067**	16 (23%)	55 (77%)	10 (21%)	38 (79%)	2 (7%)	28 (93%)	0.159

*Statistically significant values were shown in bold style*.

### Changing of Engagement Level and Learning Outcome

As the course progressed, 18 (12.1%) participants enhanced their engagement, 90 (60.4%) remained at the same level, and 41 (27.5%) became less engaged (Table 4). Compared students' grade rankings between the first and the second half of the semester, 78, 47, and 42% of each track showed the class ranking improvement, respectively (*p* = 0.030).

**Table 4:** Improve in grades ranking between first and second halves of the Pharmacology course, stratified by the change in engagement status of online video-watching pattern (*p* = 0.030 for inter-group difference by chi-square test).

**Change in engagement status of online video-watching**	**No. of students**	**No. (%) of students with improve in grades ranking**	**No. (%) of students without improve in grades ranking**
More engaged	18	14 (78%)	4 (22%)
Stationary	90	42 (47%)	48 (53%)
Less engaged	41	17 (42%)	24 (58%)

**Figure 4:**
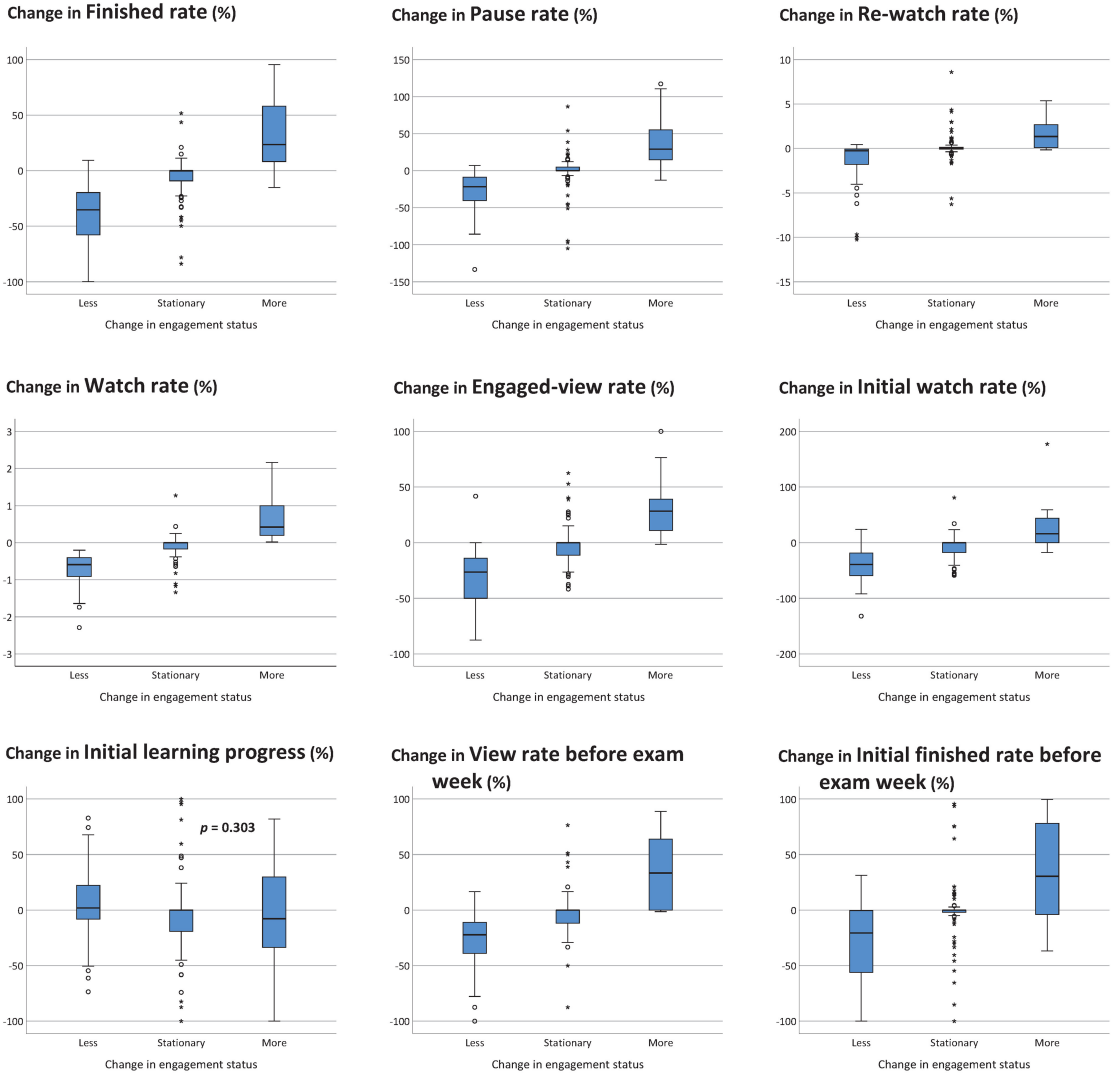
Engagement level changes and watching behavior adjustments (*p* < 0.001 for all unless otherwise mentioned). *represents extreme value; °represents potential outlier.

The changing of engagement levels implied their learning behavior adjustments. Those who became more engaged showed a better finished rate (Figure 4, left upper panel), pause rate (Figure 4, upper center panel), re-watch rate (Figure 4, right upper panel), watch rate (Figure 4, left middle panel), engaged-view rate (Figure 4, center), initial watch rate (Figure 4, right middle panel), and view rate before exam week (Figure 4, lower center panel) and initial finished rate before exam week (Figure 4, right lower panel) (all *p* < 0.001).

## Discussion

The study demonstrated three significant findings. First, the visualization plot (Figure 1) we constructed from action logs provides a very intuitive presentation on the student's engagement status in online video learning. Furthermore, using these three essential viewing behaviors, “***Completion***,” “***Pausing***,” and “***Repeated Watching***” can measure the students' engagement levels with the online video lectures effectively. Second, learning analytics revealed that the students with different levels of engagement have completely distinct online video-watching behaviors. Third, the engagement level with online video lectures is positively correlated with self-learning satisfaction, concept understanding, integrations among various subjects, and grades.

When a teacher is teaching in a physical classroom, there are plenty of visible clues to manage the class. The teacher can look for participation patterns, body language, and other non-verbal cues that give some sense of what students might be thinking. The observation and interaction are invaluable because it helps inform the teacher what the next steps are. With online learning, especially asynchronous online video lectures, this rich, analog information stream from a traditional in-person classroom has turned into a multichannel digital data stream in the virtual classroom. The data is very different, and teachers are much less experienced at interpreting it.

In a study enrolling psychology students at an Australian university, 67 participants provided 102 reasons in justifying their modality preferences about completing the class face-to-face rather than face-to-screen (48). The two themes that emerged were “more engagement” and “immediate feedback.” When an instructor teaches an online course, a great deal of time is required to learn about the learners and their needs (49). Since there is no facial expressions nor body language that can alert the teachers, teachers need constantly look for other information that can help them understand the impact of their decisions and continually ask the learners to provide feedback to know where the students are, how they feel, do they appear engaged. Teachers do need data to help them answer these questions. Therefore, teachers must encourage students to express themselves in writing as much as possible so teachers can sense whether everyone is on the same page. However, writing may not suit everyone and may be difficult for some. For example, Asian students tend to be passive learners and seldom ask questions or participate in class discussions (50–52).

Available studies on video-based learning lack sufficient understanding of how students' behaviors are related to their engagements with video content. Many studies measured engagement based on the duration of students' viewing patterns (53) or whether they navigate away from a video before completion (42). Notably, these measures focus on whether students accessed and covered different parts of the video. However, they cannot capture whether a student is actively paying attention to the video or just playing it in the background while multitasking (53). On the contrary, pausing, rewinding, or repeating videos may represent the purpose of driving attention to certain parts of the learning material (54).

By simultaneously incorporating three fundamental elements (completion, pausing, and repeated watching) of active viewing behavior, the visualization plot (Figure 1) we constructed can provide a very intuitive interpretation of the student's engagement level in online video learning. The instructor can quickly realize whether the learners have allocated enough time for self-learning (completion), generated curiosity and questions about the video content (repeated watching), and tried to resolve their questions (pausing). Furthermore, an engagement dashboard containing real-time visualizations that provide insight into how students are engaging with their online video lectures can be designed for instructors to monitor their learning status. With the intuitional presentation on the dashboard, instructors can quickly identify at-risk learners and provide timely assistance to get them back to track (55). Indeed, ways to measure students' engagement in online video lectures and blended learning courses remains an unmet need.

Though we showed that the student's engagement level was positively correlated with the learning outcome, the measurement of engagement level did not directly assess the educational objectives and learning goals, as presented in the six hierarchical levels of Bloom's Taxonomy (56, 57). In this study, more than 50% of the students had a low engagement level (either low in both the first and second half of the course, or a low-intermediate combination) and more than 50% with low engagement level answered “no” to the question “Does pharmacology meet your learning expectations.” Therefore, the teacher should worry that the engagement level may not be a good measure of learning, in particular if it can stand alone. In fact, we should not use a single dimension to measure learning effectiveness. Given that this study was designed to define engagement level by video-watching patterns and correlate engagement level with general assessment of learning outcomes, further studies on the correlation between engagement level and the achievement of different learning goals should be performed.

With the visualization plot and algorithm which we constructed to measure the engagement status of online video learning, the teachers could realize the extent of students' involvement and engagement, identify parts of the video that are difficult to understand. Therefore, the teachers can re-design video content, supplement video clips, or critically discuss the problematic parts in the next office hours. Because students in online video classes often feel more disconnected from their peers and lecturers (35), the engagement dashboard containing their engagement status and learning behaviors compared with the others can also help learners re-feel the stimulation of peers, prompt self-monitoring, and increase understanding of the learning content (55). In addition, this innovative way of assessing student's engagement during online video-based learning can be used for quality assurance purposes.

## Limitations and Further Work

- Students were required to provide feedback and response to the questionnaires in the Pharmacology course, which may introduce bias because the answers we obtain in a mandatory questionnaire can differ from optional ones.
- It is important not to judge students as active or passive learners based on the predicted level of engagement and video-watching behaviors alone (58) because watching itself can be considered an active process. On the other hand, we cannot know precisely that this “watching” occurs while the videos are playing.
- Those 149 4th-year medical students are from a top medical school in Taiwan. They should have always been serious about learning and studying hard since early in their school stage. However, they may not represent medical students in other generations. Furthermore, the findings in this study should be validated in western countries and college students other than medicine. The learning pattern in the Pharmacology course may not be the same as that in other courses. The threshold for “***Completion***,” “***Pausing***,” “***Repeated watching***” in determining the level of engagement may be different from various participants.
- The questionnaires used in this study was for the routine assessment of the quality and efficacy of teaching. They were not developed in a scientifically rigorous way. Validation of the questionnaires should be done.
- Self-efficacy, defined as the confidence to carry out the courses of action necessary to accomplish desired goals, plays an important role in influencing achievement outcomes through its dynamic interplay with environmental and behavioral determinants (59, 60). Though we recognize the importance of motivating the students to increase their self-efficacy especially for online learning, the current study is a purely observation design. We are currently planning to build an engagement dashboard on the NTU COOL platform. We look forward to seeing whether the learning behaviors are changed and learning outcomes are improved by the engagement dashboard.

## Conclusion

Since online video-based learning is more common in higher education, learning analytics is essential for instructors to understand students' engagement situations and evaluate teaching effectiveness for continuous improvements.

This study developed the pre-defined thresholds and algorithms on how the pre-clinical medical students' video viewing logs of online Pharmacology course can be analyzed and visualized more intuitively to present their learning situations. Furthermore, the high, intermediate, and low engagement level with online video-based lectures was defined by the composition of different learning behaviors of completion, pausing, and repeated watching.

The study results showed that the highly engaged students had a better learning outcome, higher learning satisfaction and were more beneficial in knowledge construction and integration.

Unlike in-person or hybrid courses, instructors of online video-based learning cannot interact with students in real-time. Therefore, the article suggested that an engagement dashboard containing real-time visualized information on each students' video-based learning situations can be designed, developed, and provided for instructors to monitor students' learning progress, evaluate teaching effectiveness, improve course content, adjust teaching styles, identify and assist students at risk in a timely fashion.

The engagement dashboard containing students' engagement status and learning behaviors compared with the others can also be customized and provided for each student to re-feel the stimulation of peers, prompt self-monitoring, improve their learning attitudes and disciplines for better learning outcomes.

## Data Availability Statement

The datasets used and analyzed during the current study are available from the corresponding author upon reasonable request.

## Ethics Statement

The studies involving human participants were reviewed and approved by Research Ethics Committee (REC) of National Taiwan University Hospital (NTUH), Taipei, Taiwan. Written informed consent for participation was not required for this study in accordance with the national legislation and the institutional requirements.

## Author Contributions

J-YW and C-HY drafted the manuscript. J-YW, B-CS, and Y-HN designed the study. J-YW, W-CL, K-CY, and I-WC conducted data processing. J-YW, W-CL, K-CY, I-WC, and B-CS performed data analysis. Y-HN was the director responsible for general organization and instruction. All authors contributed to the article and approved the submitted version.

## Funding

The study was supported by the High Education Sprout Project of the Ministry of Education (NTU-109L3901; NTU-110L3901). The funders had no role in the study design, data collection and analysis, decision to publish, or manuscript preparation.

## Conflict of Interest

The authors declare that the research was conducted in the absence of any commercial or financial relationships that could be construed as a potential conflict of interest.

## Publisher's Note

All claims expressed in this article are solely those of the authors and do not necessarily represent those of their affiliated organizations, or those of the publisher, the editors and the reviewers. Any product that may be evaluated in this article, or claim that may be made by its manufacturer, is not guaranteed or endorsed by the publisher.
