# Supplementary material for: Highly Engaged Video-Watching Pattern in Asynchronous Online Pharmacology Course in Pre-clinical 4th-Year Medical Students Was Associated With a Good Self-Expectation, Understanding, and Performance
Source: Front Med (Lausanne). 2022 Jan 21;8:799412. doi: 10.3389/fmed.2021.799412 (PMC8814113; doi:10.3389/fmed.2021.799412)

**SUPPLEMENTARY FILE**

**Highly engaged video-watching pattern in asynchronous online pharmacology course in pre-clinical 4^th^-year medical students was associated with a good self-expectation, understanding, and performance**

**CURRICULUM DEVELOPMENT PROCESS OF NATIONAL TAIWAN UNIVERSITY COLLEGE OF MEDICINE (NTUCM)**

The vision of NTUCM is to ultimately become the benchmark of medical education and bio-med research in Asia and to be one of the top medical colleges worldwide. To align with the vision and mission, NTUCM has organized different functional units in the teaching, research, and administration sectors to facilitate collaboration efficiency and operation quality. The organization structure is illustrated in **Figure S1**.

After passing the college entrance exam from senior high school, medical students in Taiwan will receive a 6-year education. The course structure of medicine consists of general education courses in the first and second years, and integrated courses between basic science and clinical disciplines in the third and fourth years, followed by two clinical years in the university-affiliated medical centre.

**1. The Curriculum Committee and The Center of Faculty Development**

To improve teaching quality and enhance students’ learning experiences, the Curriculum Committee of NTUCM continuously reviews and revises the course design as well as the curriculum structure as a whole.

However, medical practices across different schools and departments span widely. To ensure the instructors can deliver fundamental medical knowledge in both horizontal and vertical integration approaches, a supporting office, the Office of Medical Education, established in 1995, was renamed the Center of Faculty Development (CFD) in 2010. Currently, there are five divisions under the CFD: 1) Curriculum Integration; 2) Problem-based Learning Curriculum; 3) Teaching Evaluation; 4) Research and Development, and; 5) General Education.

**2. Curriculum Integration**

Curricular integration has become a popular program in the reorganization of modern medical education (1, 2). Both vertical (a science course taught by various instructors across different academic years) and horizontal (several courses in different medical domains taught in one academic year) integration of undergraduate medical curriculum have been shown effective for problem-based and lifelong learning (3, 4).

The CFD of NTUCM has started to construct an integrated organ system-based curriculum for senior undergraduate medical students since 1992. By the end of the 2015/2016 academic year, the curriculum has been well-organized to help the students exploit conceptual awareness, integrate different ideas, and build their medical knowledge (**Figure S2**).

**3. Instructional design principle of pharmacology course**

Our curriculum design and continuous improvement model is ADDIE, a well-known 5-step process widely used by instructional designers (**Figure S3**) (5). For curriculum improvement, the CFD uses different platforms to collaborate with the Curriculum Committee, the instructors, and the students from different programs to ensure communication efficiency and effectiveness for a tangible result. A fit-gap analysis workshop is regularly conducted to review the learning objectives, identify the current status and the gaps. The issues reported from the students’ oral feedbacks, obtained from the written questionnaires, or reflected by the instructors will also be addressed in the fit-gap analysis workshop. After drafting the curriculum or course plan and selecting suitable teaching media and methods, the proposal is submitted to the Curriculum Committee for critical review. According to the review comments, the proposal might have to be modified and resubmitted several times.

After the course proposal is approved and offered, all the related information, including syllabus, teaching notes, and assessment results will be recorded in the Collaborative Enhanced Instruction By Asynchronous Learning system (CEIBA), a web-based course management system built by NTU to facilitate the communication between teachers and students.

**References**

1. Brauer DG, Ferguson KJ. The integrated curriculum in medical education: AMEE Guide No. 96. Med Teach. 2015;37(4):312-22.

2. Drake RL, Pawlina W. Multimodal education in anatomy: The perfect opportunity. Anat Sci Educ. 2014;7(1):1-2.

3. Brynhildsen J, Dahle LO, Behrbohm Fallsberg M, Rundquist I, Hammar M. Attitudes among students and teachers on vertical integration between clinical medicine and basic science within a problem-based undergraduate medical curriculum. Med Teach. 2002;24(3):286-8.

4. Furmedge DS, Smith LJ, Sturrock A. Developing doctors: what are the attitudes and perceptions of year 1 and 2 medical students towards a new integrated formative objective structured clinical examination? BMC Med Educ. 2016;16:32.

5. Dick W, Carey L. The systematic design of instruction. 4 ed. New York: Harper Collins College Publishers; 1996.

**Table S1.** Questionnaires on learning efficacy and satisfaction

|  | **Strongly disagree** | **Disagree** | **Neutral** | **Agree** | **Strongly agree** |
| --- | --- | --- | --- | --- | --- |
| Does Pharmacology meet your learning expectations? | **□** | **□** | **□** | **□** | **□** |
| Do the organized course videos help you understand the underlying concepts? | **□** | **□** | **□** | **□** | **□** |
| Are you fine with the difficulty level? | **□** | **□** | **□** | **□** | **□** |
| Can you connect Pharmacology with other classes? | **□** | **□** | **□** | **□** | **□** |
| Can you use what you've already learned from Pharmacology to construct the understandings of other subjects? | **□** | **□** | **□** | **□** | **□** |

**Table S2.** Students’ response of questionnaires

|  | **First half of the course** | **Second half of the course** |
| --- | --- | --- |
| Does Pharmacology meet your learning expectations?  Strongly agree  Agree  Neutral  Disagree  Strongly disagree | 5 (3.4%)  52 (34.9%)  87 (58.4%)  2 (1.3%)  3 (2%) | 7 (4.7%)  61 (40.9%)  78 (52.3%)  1 (0.7%)  1 (0.7%) |
| Do the organized course videos help you understand the underlying concepts?  Strongly agree  Agree  Neutral  Disagree  Strongly disagree | 26 (17.4%)  73 (49.0%)  47 (31.5%)  2 (1.3%)  1 (0.7%) | 19 (12.8%)  78 (52.3%)  44 (29.5%)  2 (1.3%)  5 (3.4%) |
| Are you fine with the difficulty level?  Strongly agree  Agree  Neutral  Disagree  Strongly disagree | 33 (22.1%)  83 (55.7%)  30 (20.1%)  2 (1.3%)  1 (0.7%) | 19 (12.8%)  78 (52.3%)  44 (29.5%)  2 (1.3%)  5 (3.4%) |
| Can you connect Pharmacology with other classes?  Strongly agree  Agree  Neutral  Disagree  Strongly disagree | 33 (22.1%)  88 (59.1%)  25 (16.8%)  2 (1.3%)  1 (0.7%) | 36 (24.2%)  84 (56.4%)  26 (17.4%)  0 (0%)  2 (1.3%) |
| Can you use what you've already learned from Pharmacology to construct the understandings of other subjects?  Strongly agree  Agree  Neutral  Disagree  Strongly disagree | 31 (20.8%)  86 (57.7%)  29 (19.5%)  2 (1.3%)  1 (0.7%) | 37 (24.8%)  83 (55.7%)  26 (17.4%)  0 (0%)  2 (1.3%) |

**FIGURE LEGENDS**

**Figure S1.** Organization chart of the National Taiwan University College of Medicine

* Complete list of the department is not shown.

**
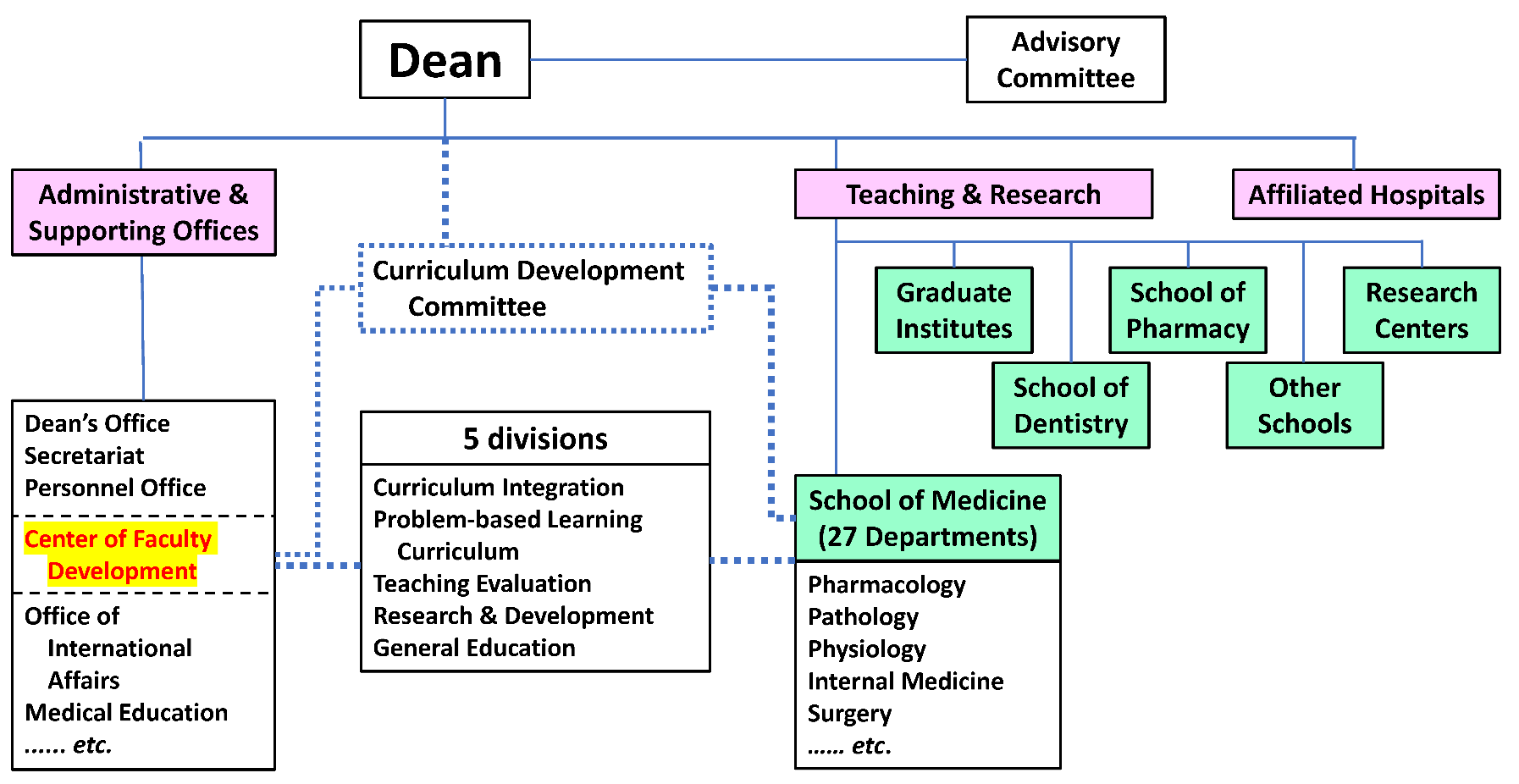
**

**Figure S2.** Curriculum integration between basic science and clinical disciplines for the 4^th^-year medical students of the 6-year course in 2020 Spring





**Figure S3.** The curriculum design principle

Abbreviation: CC, Curriculum Committee; CFD, Center of Faculty Development; CID, Curriculum Integration Division; GED, General Education Division; NTU CEIBA, Collaborative Enhanced Instruction By Asynchronous Learning system of National Taiwan University; PBLCD, Problem-based Learning Curriculum Division; RDD, Research and Development Division; TED, Teaching Evaluation Division.

CID, GED, PBLCD, RDD, and TED were the five divisions of the CDF.

* Regular activity, twice per semester.

^#^ Regular activity, five times per semester.


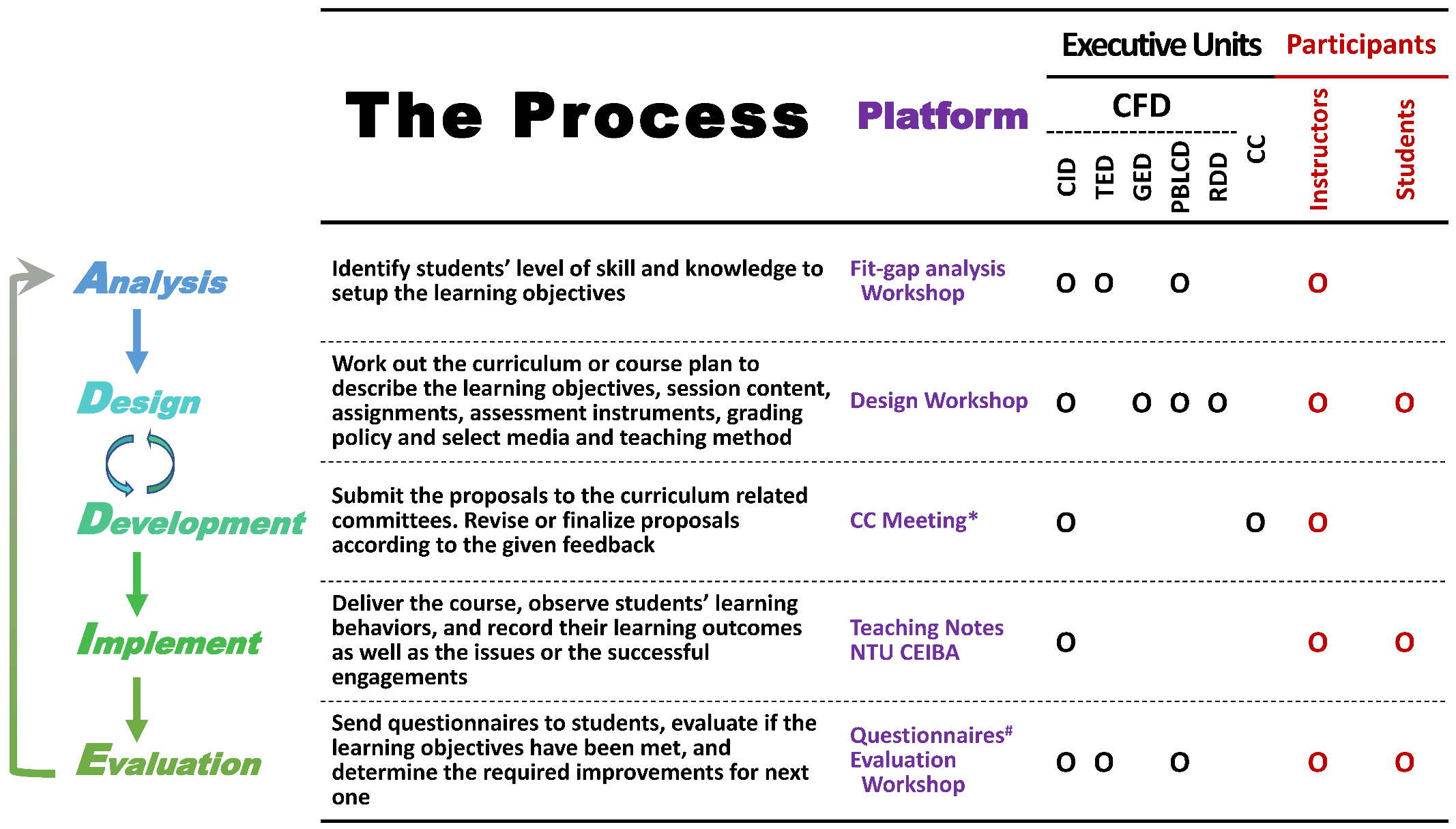


**Figure S4.** Screenshots to illustrate the NTU COOL learning platform

Step 1: Login


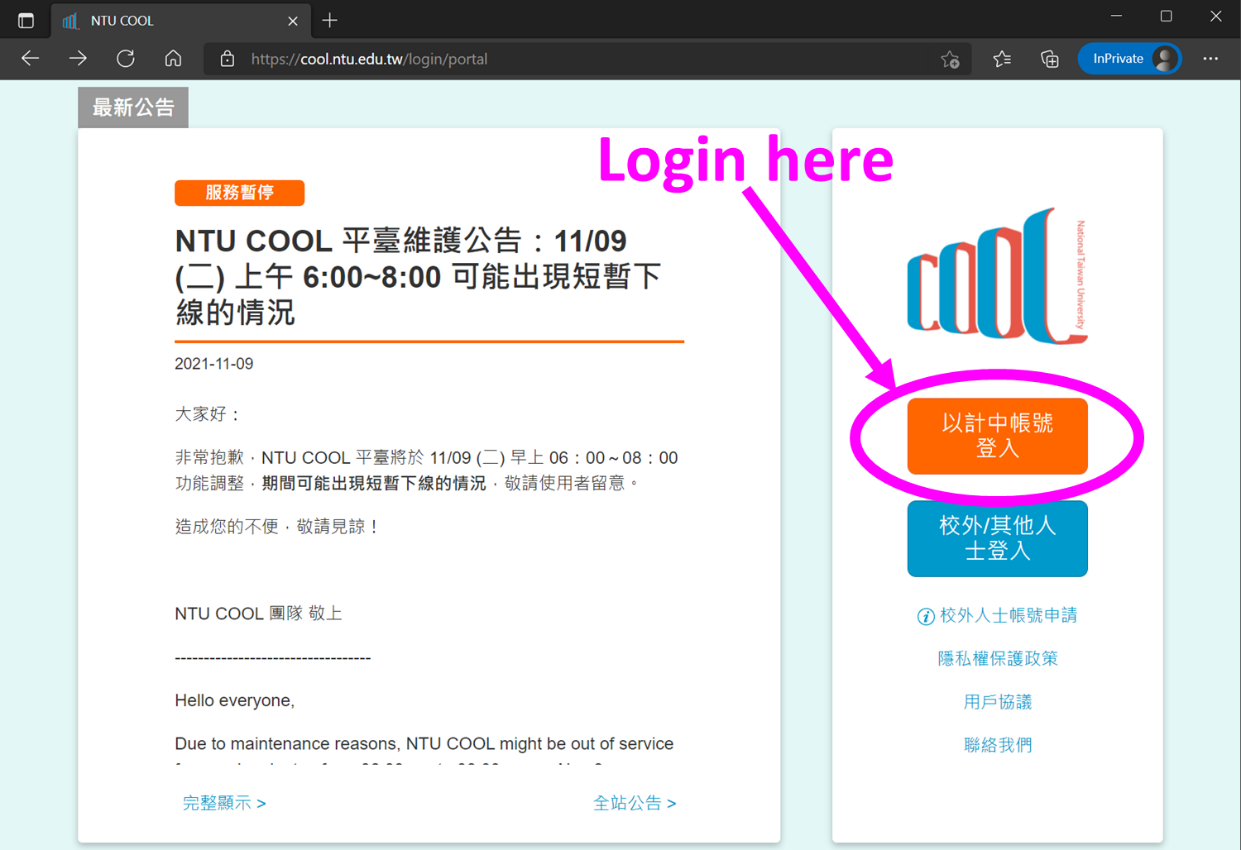


Step 2: Enter account and password


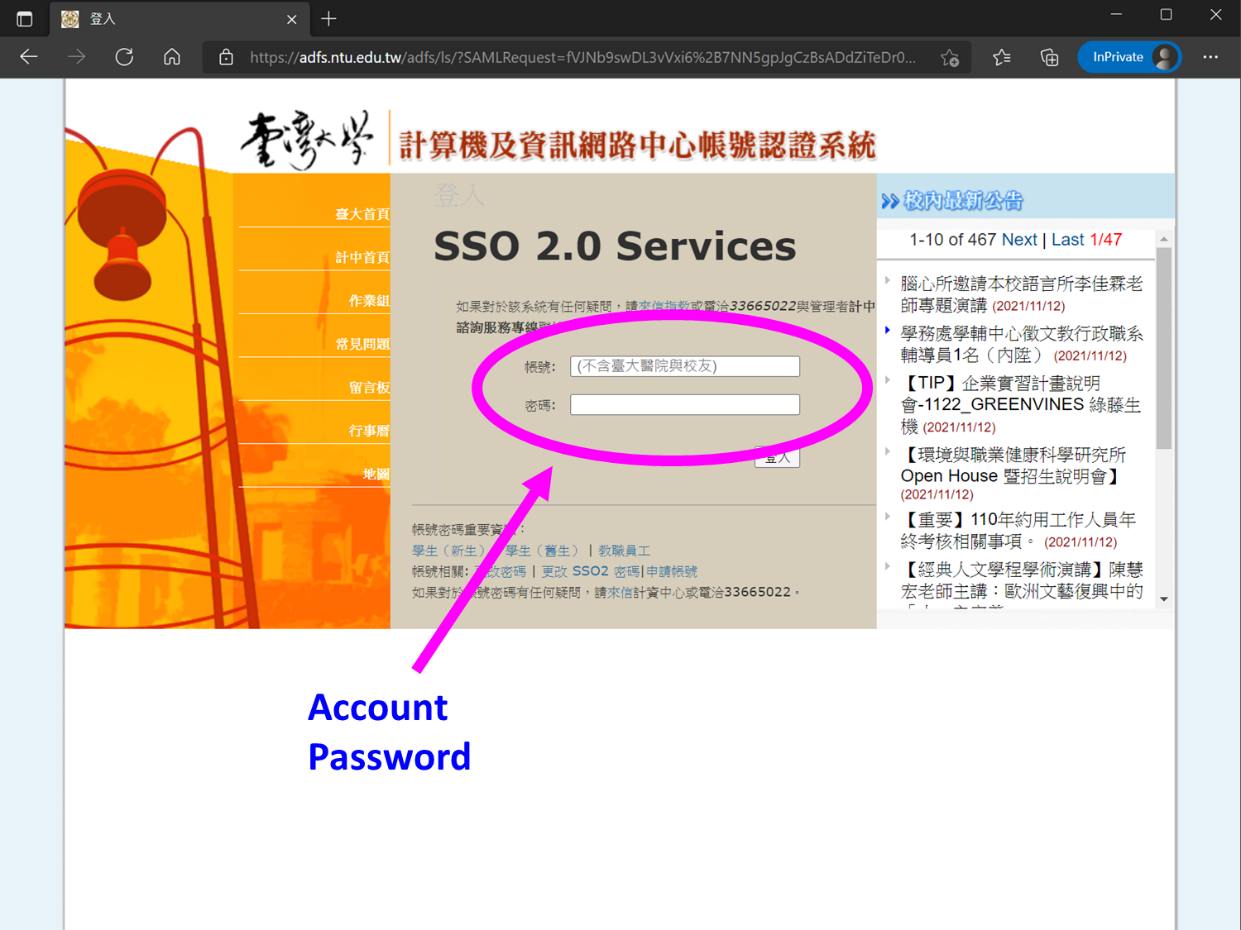


Step 3. Integrated organ system-based scaffolding learning modules


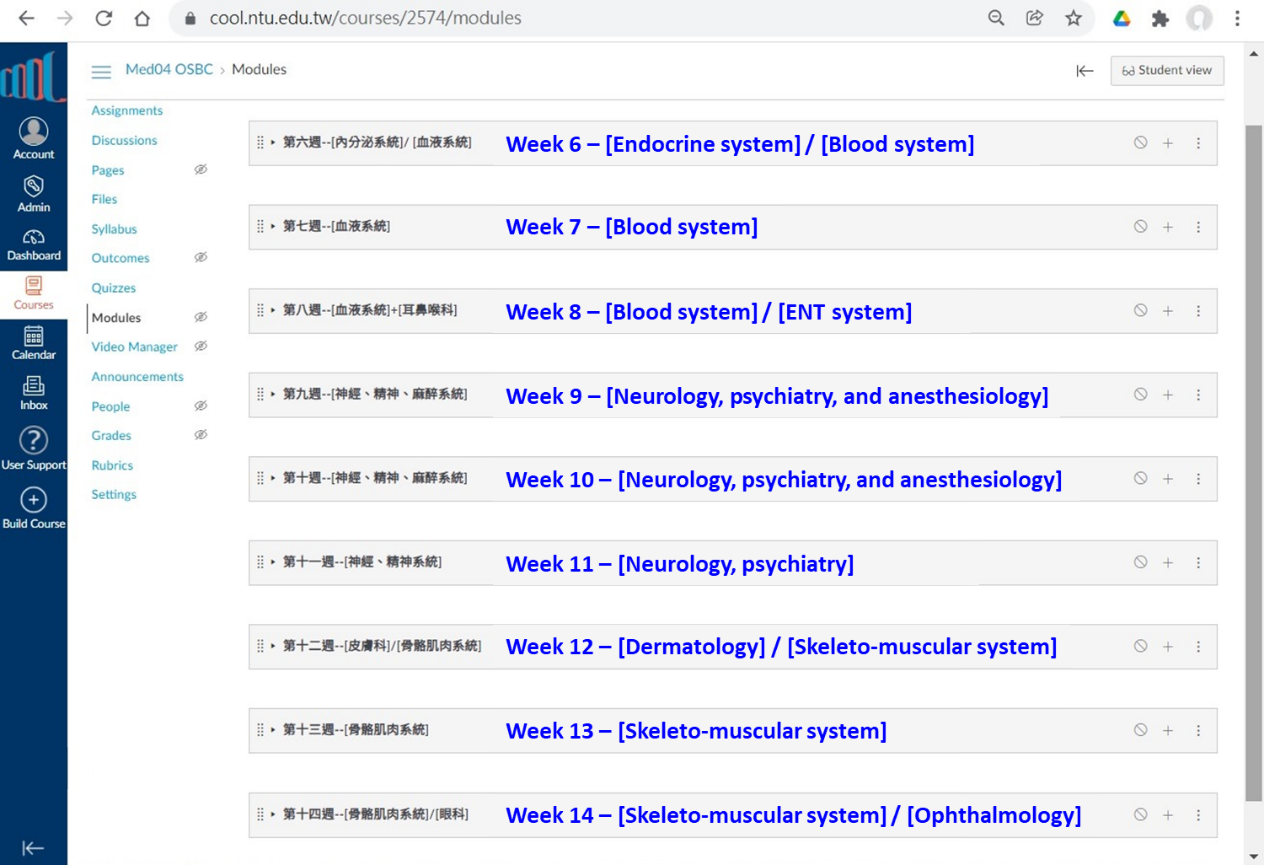


Step 4. Course content when clicking on specific week


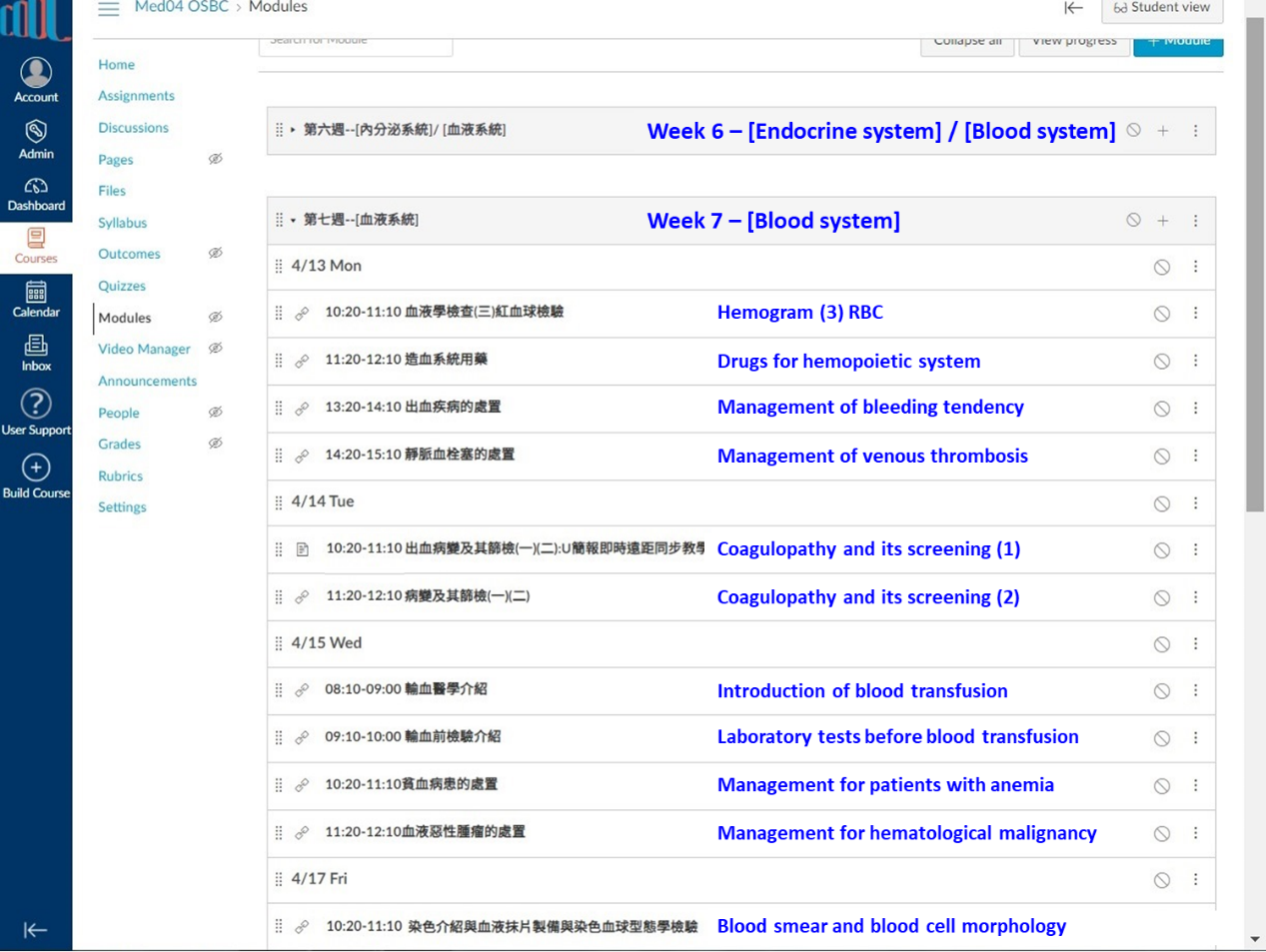


Step 5. Watch video and start an interactive discussion


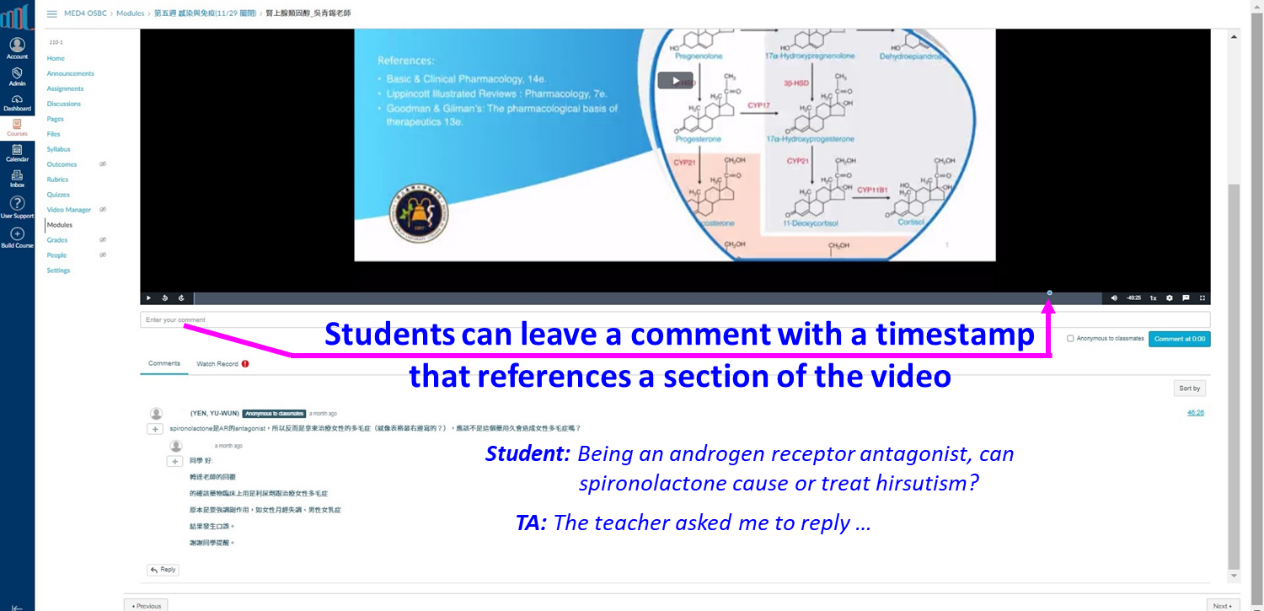

Supplement: Supplementary file 1 [file Data_Sheet_1.DOCX]
